# Graphic Novel for Patients Affected by Pancreatic Lesions Undergoing Endoscopic Ultrasound with Fine Needle Biopsy: A Pilot Randomized Study

**DOI:** 10.3390/healthcare14060699

**Published:** 2026-03-10

**Authors:** Giacomo Emanuele Maria Rizzo, Giuseppe Infantino, Fabio Tuzzolino, Mario Traina, Giovanni Di Piazza, Daniele La Milia, Gabriele Rancatore, Lucio Carrozza, Dario Quintini, Dario Ligresti, Margherita Pizzicannella, Nicoletta Belluardo, Elio D’amore, Giuseppe Rizzo, Cinzia Di Benedetto, Ugo Palazzo, Ilaria Tarantino

**Affiliations:** 1 Gastroenterology and Endoscopy Unit, IRCCS-ISMETT (Mediterranean Institute for Transplantation and Advanced Therapies), 90127 Palermo, Italymtraina@ismett.edu (M.T.);; 2 University of Pittsburgh Medical Center Italy (UPMC Italy), 90127 Palermo, Italy; 3 Statistics and Data Management Services, IRCCS-ISMETT (Mediterranean Institute for Transplantation and Advanced Therapies), 90127 Palermo, Italy; 4 Healthcare Management, IRCCS-ISMETT (Mediterranean Institute for Transplantation and Advanced Therapies), 90127 Palermo, Italy

**Keywords:** endoscopic ultrasound, EUS, graphic novel, pancreas, anxiety, patient care

## Abstract

**Background/Objectives**: Endoscopic Ultrasound with Fine Needle Biopsy (EUS-FNB) of pancreatic lesions often induces patient anxiety. Graphic medicine, an emerging health communication tool, could potentially mitigate this. This pilot study aimed to explore the feasibility of a graphic novel in reducing anxiety in adult patients awaiting EUS-FNB. **Methods**: This prospective, single-center, randomized pilot study was conducted from June 2024 to March 2025 in patients aged 18–89 years. The intervention group received a comic panel detailing the EUS-FNB routine, while controls had standard care. Anxiety was measured using the Beck Anxiety Inventory (BAI) and modified DASS-21 (mASS-14). **Results**: Overall, 65 patients (33 “Graphic Novel”, 32 “Control”) were included. Mean BAI was 4.88 (graphic novel) vs. 7.25 (controls, *p* = 0.092), and mASS-14 was 4.97 vs. 6.22 (*p* = 0.261). Anxiety prevalence was low (4.6% BAI, 13.8% mASS-14). Controls were more symptomatic (69.2%) and had a higher rate of pancreatic cancer (*n* = 20) compared to the graphic novel group (*n* = 6). Subgroup analyses showed that BAI was slightly lower for patients with children and no prior surgical experience when using graphic novels. Trends for lower anxiety appeared in those on chronic medication, under surveillance, or with solid/suspected metastatic lesions. **Conclusions**: This pilot study suggests that a graphic novel may help to reduce anxiety and stress scores in patients undergoing diagnostic procedures for pancreatic lesions. However, it needs confirmation in larger, adequately powered trials.

## 1. Introduction

Endoscopic ultrasound (EUS) is the principal modality for the diagnosis and treatment of severe, and occasionally life-threatening, pancreatic disorders. Pancreatic cancer currently ranks as the third leading cause of cancer death in the United States, with an estimated 67,530 new cases and 52,740 deaths projected for 2026, underscoring the critical need for timely and accurate characterization of pancreatic lesions suspicious for malignancy in order to improve patient outcomes [1]. In the context of pancreatic lesions, diagnostic procedures such as EUS with fine needle biopsy (EUS-FNB) can induce states of fear and anxiety. Using nationwide claims data from 22,863 newly diagnosed cancer patients in Japan, the crude incidence rates for depression and anxiety were 3.36 and 3.11 per 1000 person-months, respectively [2]. Anxiety peaked in the month of cancer diagnosis, depression peaked within the first three months thereafter, and pancreatic cancer exhibited the highest incidence of both disorders, findings that underscore the substantial psychological burden surrounding the diagnostic phase in patients with suspected pancreatic malignancy [2]. Therefore, the apprehension and/or anxiety associated with the biopsy may be disproportionate relative to the actual risks or objective threat [3]. Waiting for a biopsy increases fear, given that a cancer diagnosis is arguably one of the most stressful, life-altering events an individual can encounter [4]. Graphic medicine is a new subfield of research and practice [5], defined as a form of visual storytelling exploring narratives in health care, cancer, healing, and disability [6]. Graphic medicine is currently a trending topic in health communication [7], and researchers are showing growing interest in it as an effective communication tool [8,9], even for health and medical education and teaching [10,11]. Graphic medicine may reduce pre-procedural anxiety by decreasing uncertainty about what will happen, setting realistic expectations regarding the procedure, and enhancing patients’ perceived coping self-efficacy, through concrete visual narratives that depict the step-by-step experience of EUS-FNB and normalize common emotional reactions [12,13]. With their images, limited text, and relatively short format, comics provide an easy option for the effective communication of health-related messages [14]. Consequently, it has been applied to several fields with different aims, such as an educational tool in medicine to improve knowledge, support prevention campaigns, and promote health-seeking behaviours in conditions like cervical cancer [15], HIV infection [16], and chronic liver disease [17], as well as anxiety-focused interventions in patients awaiting invasive procedures, such as oral biopsy for potentially malignant disorders [18]. To our knowledge, no studies have evaluated graphic novels in endoscopy as a pre-procedural communication tool to address anxiety and stress in patients undergoing EUS-FNB, so this pilot randomized study was designed as a hypothesis-generating study for exploring the feasibility and potential impact of a graphic novel intervention in this specific setting.

## 2. Materials and Methods

### 2.1. Study Design

This was a prospective single-center pilot study with primarily exploratory aims evaluating the influence of graphic novels on the stress levels and behavioural responses of patients with pancreatic lesions undergoing EUS-FNB, following CONSORT guidelines [19] and including the TIDieR (Template for Intervention Description and Replication) [20] (Supplementary Materials). The study was conducted from June 2024 to March 2025. Written informed consent was obtained from all participants prior to enrolment, and the research was conducted in accordance with the principles of the Declaration of Helsinki. The protocol was approved by our ethical committee (IRRB/43/23) and registered to ClinicalTrials.gov (NCT06268106). The primary outcome was to obtain preliminary estimates of post-procedural anxiety, as measured by the Beck Anxiety Inventory (BAI, Figure S1) [21], in the two groups, together with their variability. The secondary outcomes were exploratory and included the descriptive estimation of mean scores and variability using a modified version of the Depression Anxiety Stress Scales-21 (mDASS-21, Figure S2) [22], and to explore whether the effect of the intervention differed across patient-related characteristics (exploratory analyses of effect modification). The presence of anxiety was defined as a BAI score higher than 16 (moderate or severe) or mDASS-21 score higher than 10 (Moderate or severe or extremely severe).

### 2.2. Inclusion and Exclusion Criteria

All patients with radiological and clinical suspicion of a pancreatic lesion were evaluated for the following inclusion criteria: (1) age ≥ 18 years; (2) pancreatic solid mass or partially solid in the case of a cystic component requiring tissue acquisition; (3) the ability to read and understand the informed consent form; (4) patients not affected by any known psychological disorder. Exclusion criteria were (1) patients with cognitive deficits, such that they cannot adequately complete the questionnaire, and the visually impaired; (2) pregnancy; (3) patients using benzodiazepines or other psychotropic medications; (4) patients with a previous diagnosis of cancer.

### 2.3. Pilot Study Procedure

Following hospital admission and after confirmation of eligibility and written informed consent, patients were randomly allocated to the intervention or control group using a computer-generated randomization sequence created by an independent statistician using Random Allocation Software (version 1.0, Isfahan University of Medical Sciences, Iran). Simple randomization with a 1:1 allocation ratio was employed without stratification or blocking. The randomization assignment was communicated to the physician, acquiring consent only after patient eligibility had been confirmed and consent obtained, ensuring allocation concealment until the point of randomization. An officer who was the only person with protected access to the randomization list ensured the allocation concealment. Due to the nature of the intervention, neither patients nor clinical staff could be blinded to group allocation. The control group experienced the standard daily routine for EUS-FNB of pancreatic lesions at our institute, including procedure explanation and consent acquisition. It included an initial colloquium with the physician and nurse explaining how the day would unfold, addressing any procedure-related and medical care-related questions the patient may have. The patient then waits in their room until it is their turn in order to be brought to the endoscopic room for performing EUS-FNB. In case of randomization to the test group (Figure S3), the graphic novel was provided to all patients after the initial colloquium, leaving them waiting for the procedure with the graphic panel, allowing them to read it and to more gradually assimilate how their day would unfold [23]. All EUS-FNB procedures were performed under general anaesthesia or deep sedation according to the institutional protocol.

### 2.4. Graphic Novel—Comic Panel

**Figure 1:**
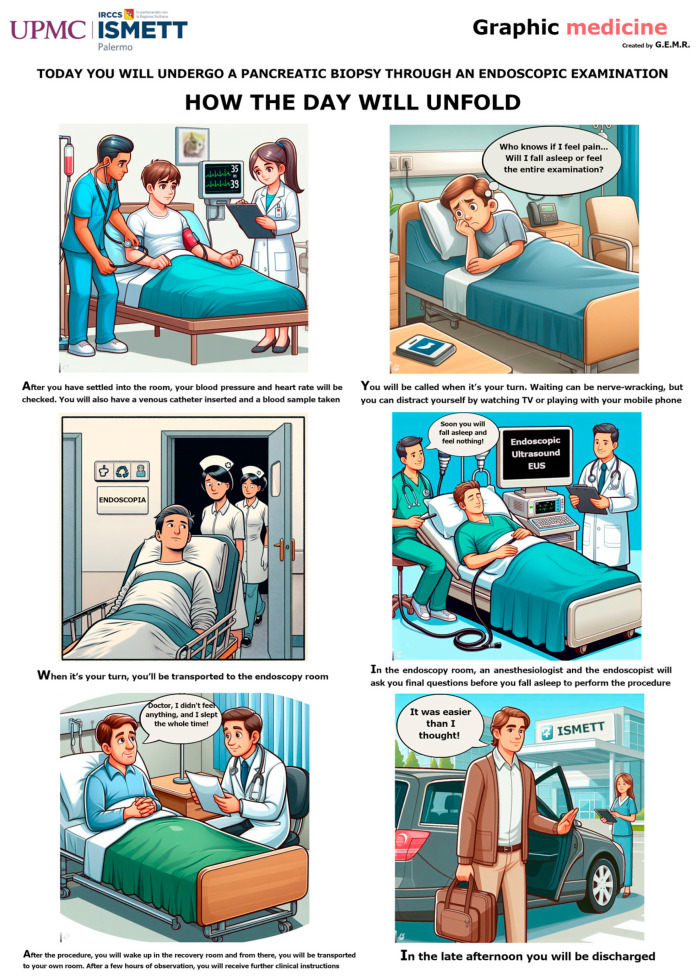
The graphic novel administered to patients randomized to the interventional group.

The authors created a comic panel consisting of a sequence of 6 colourful vignettes in which the routine procedure of EUS-FNB is described (Figure 1). The initial drafts of the six vignettes were created using artificial intelligence (AI image creator using DALL-E3 technology) and later modified by one author (G.E.M.R.) using open-source graphic software (GIMP, GNU Image Manipulation Program, Version 2.10.28, gimp.org). Figure 1 provides a structured overview of the graphic novel intervention, which consists of six sequential panels covering: the hospital setting and arrival, pre-procedure preparation, positioning in the endoscopy room, steps of EUS-FNB, the immediate post-procedure recovery, and discharge of the patient. The panel was designed to be readable in approximately 3–5 min by adults with a basic to intermediate literacy level, using simple language and clear visual cues. We did not formally measure whether patients read the entire panel, time spent, or comprehension, so this limitation will be addressed in future studies.

### 2.5. Assessing Anxiety

After EUS-FNB, all recruited patients received the BAI [21] and the mDASS-21 [22], which are more deeply explained in Supplementary Materials. Due to the nature of the graphic novel intervention, neither patients nor clinical staff could be blinded to group allocation, as in the previous phases. A physician, nurse, or clinical assistant administered the questionnaires, asking for questions and clarification. The BAI is a 21-question self-report inventory used to assess anxiety severity in adults, taking approximately 5 to 10 min to be completed. Respondents answer questions about common anxiety symptoms, such as numbness, tingling, sweating unrelated to heat, and fear of catastrophic events. Scores range from 0 (not at all) to 3 (severe), with higher scores indicating more severe anxiety. Standardized cut-offs classify anxiety levels as minimal (0–7), mild (8–15), moderate (16–25), or severe (26–63). The Italian version was validated by Sica & Ghisi (2007, The Italian Versions of the Beck Anxiety Inventory and the Beck Depression Inventory-II: Psychometric Properties and Discriminant Power) [24], demonstrating good to excellent internal consistency ([Cronbach Index] α = 0.87–0.90) and strong discriminant validity in non-clinical samples (*n* = 1485) and anxious patients. The other questionnaire is a modified version of the DASS-21, which originally consisted of 21 items divided into three subscales (anxiety, stress, and depression) with seven items each [25]. We focused on the anxiety and stress subscales [26], removing the depression subscale. Many studies have demonstrated that the Depression, Anxiety, and Stress subscales of the DASS-21 show good internal consistency, factorial validity, and convergent validity when used as separate measures, supporting the independent use of the anxiety and stress subscales while maintaining robust psychometric properties [27,28,29,30,31]. Specifically, the anxiety subscale assesses physiological arousal, situational anxiety, and subjective experience of the effects of anxiety, while the stress subscale evaluates chronic non-specific arousal, difficulty in relaxing, nervous tension, irritability, agitation, impatience, and overactivity. Each item is rated on a 4-point scale from 0 to 3. The total score ranges from 0 to 42 in our modified version, with higher scores indicating higher levels of anxiety and stress. Brown et al. [27] confirmed excellent internal consistency for DASS-21 (anxiety α = 0.89, stress α = 0.93). We named our modified version mDASS-21 or mASS-14. Both questionnaires were administered in their Italian versions to avoid language barriers (English version in Figures S1 and S2).

### 2.6. Data Collection

Data were anonymously collected prospectively in a dedicated electronic case report form (eCRF) through a secure platform (RedCap), and included demographic data, level of education, family status, clinical presentation, and lesion characteristics. Medical history, including previous clinically relevant conditions, such as hospitalizations, surgeries, chronic medical therapies, or familiarity with cancer, was also collected.

### 2.7. Statistical Analysis

Given the pilot nature of the study and the absence of a formal power calculation, analyses were considered exploratory, and emphasis was placed on effect size estimates and confidence intervals rather than on *p*-values as confirmatory evidence. Continuous variables were assessed for normality of distribution using the Shapiro–Wilk test and visual inspection of Q-Q plots. Based on the results of normality testing, appropriate parametric (independent samples *t*-tests) or non-parametric tests were selected for between-group comparisons. Chi-square tests were performed to examine associations between the categorical versions of the scores and the treatment groups, while Fisher’s exact test was used when expected cell counts were small, and the chi-square approximation was not reliable. For investigating the effects of the treatment and characteristics of patients at baseline and characteristics of the pancreatic lesions, mASS-14 and BAI score, generalized linear models (GLMs) with interaction terms and Tukey-adjusted least squares means comparisons were applied. This approach allows for both mean comparisons and the modelling of interactions and covariates. Baseline balance between groups was summarised using standardized mean differences (SMDs) for key continuous and binary variables [32]. Subgroup analyses were based on different variables. Additional GLMs were used to assess the influence of intercurrent time on both mASS-14 and BAI scores. Notably, the statistician performing data analysis was not blinded to group assignment. The primary analysis consisted of unadjusted comparisons of mean BAI and mASS-14 scores between the two randomized groups. GLMs with interaction terms and multiple subgroup analyses were conducted in a secondary, exploratory manner to generate hypotheses about potential effect modifiers, and all *p*-values from these models should be interpreted as exploratory. All statistical analyses were performed using SAS software (v 15.2, SAS Institute Inc., SAS Campus Drive, Cary, NC, USA).

## 3. Results

### 3.1. Participants’ Characteristics

A total of 65 patients (mean age 64.5 [±13.6] years) were included, 33 randomized to the “Graphic Novel” group and 32 to the “Control” group. Demographic and clinical characteristics were comparable between groups (Table 1). The comorbidity index was 3.4 (1.6), and the most common educational level was high school degree (*n* = 28, 43.1%). Twenty-nine (44.6%) patients were taking long-term drugs for chronic conditions. Pancreatic lesions were solid in 44 (66.7%) of cases, cystic in 17 (26.2%), and mixed (solid-cystic) in 4 (6.3%) (*p* = 0.195, Table 2). The mean time from radiological evidence of pancreatic lesion to EUS-FNB (defined “*intercurrent time*”) was 36.3 ± 35.2 days, with minimal differences between the two arms (Delta [Δ] = −1.22, CI95% −3.3–0.88 days [*p* = 0.25] for mASS-14 and Δ = −2.35, CI95% −4.97–0.27 days [*p* = 0.08], for BAI).

### 3.2. Anxiety Assessment

**Figure 2:**
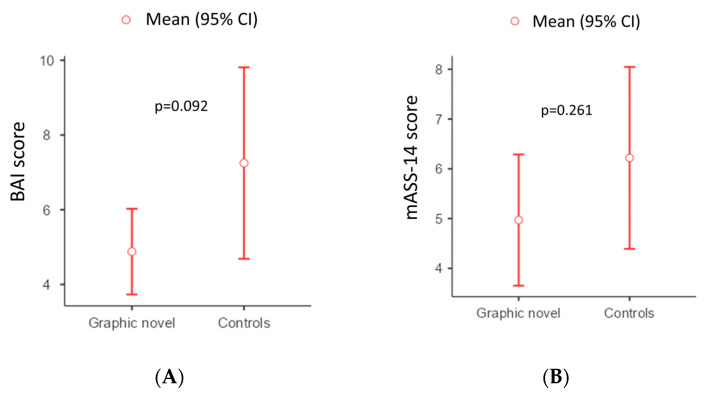
Graphical view of the differences between the two arms according to mASS-14 (**A**) and BAI (**B**) scores.

Overall, the mean BAI score was 6.05 (±5.58), and the mean mASS-14 score was 5.58 (±4.45). The “Graphic Novel” group showed a mean BAI score of 4.88 (±3.24), compared to a mean of 7.25 (±7.11) in the “Control” group, with a mean difference of −2.37 (95% CI, −5.1 to 0.4, *p* = 0.092) (Figure 2A). The “Graphic Novel” group showed a mean mASS-14 score of 4.97 (±3.72), compared to a mean of 6.22 (±5.07) in the “Control” group, with a mean difference of −1.25 (95% CI, −3.45 to 0.95, *p* = 0.261) (Figure 2B). The BAI showed excellent internal consistency (α = 0.858), while it was acceptable (α = 0.771) for mASS-14.

Differences in the use of graphic novels did not reach statistical significance for both the BAI and mASS-14 outcome (*p* = 0.092 and *p* = 0.261, respectively, Table 3).

**Figure 3:**
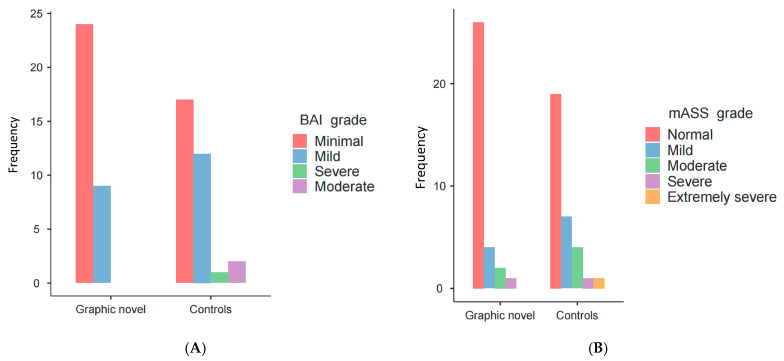
Graphical view of the level of the anxiety/stress between the two arms according to BAI (**A**) and mASS-14 (**B**) scores.

The prevalence of anxiety, evaluated according to BAI and mASS-14 score, was 4.6% and 13.8%, respectively. Level of anxiety was mainly minimal according to BAI in both the graphic group (*n* = 24, 58.5%) and the controls (*n* = 17, 41.5%, Figure 3A), and similarly it was mainly normal according to mASS-14 both in the graphic novel group (*n* = 26, 78.8%) and in the controls (*n* = 19, 59.4%, Figure 3B).

### 3.3. Clinical Presentation

Clinical presentation was different between the two arms, with a higher frequency of symptoms in the control arm (*n* = 18, 69.2%) and a higher frequency of incidental diagnosis in the graphic novel arm (*n* = 23, 65.7%; *p* = 0.026, adjusted [Adj] *p* = 0.223). The latest result was deeper analysed with adjustment for multiple comparisons when evaluating differences in the score of BAI and mASS-14. Specifically, the BAI score was higher in symptomatic controls compared to patients with incidental diagnosis (*p* = 0.026, Adj-*p* = 0.223) or in the surveillance program (*p* = 0.026, Adj-*p* = 0.228) in the graphic novel arm. The mASS-14 score was higher in the symptomatic controls compared to incidental patients (*p* = 0.084, Adj-*p* = 0.512), while it was higher compared to patients in the surveillance program in the graphic novel (*p* = 0.034, Adj-*p* = 0.034).

### 3.4. Subgroup Analysis

Among patients in the graphic novel (GN) arm, patients with first-degree relatives (*n* = 3) and second-degree relatives (*n* = 2) had differences in scores (BAI = 3.33 vs. 9.50, Adj-*p* = 0.0066; mASS-14 = 3.33 vs. 8.0, Adj-*p* = 0.1113). In the second-degree relative group (*n* = 4), controls (*n* = 2) showed lower scores compared to the GN group (BAI = 1.0 vs. 9.5, Adj-*p* = 0.0003; mASS-14 = 2.0 vs. 8.0, Adj-*p* = 0.0419) (Figure S4). Among patients with children (*n* = 59), the GN group shows a lower BAI score (BAI = 4.66) compared to controls (BAI = 7.63, Adj-*p* = 0.134) (Figure S5). Controls showed no differences between those who had undergone surgery and those who had not, whereas among patients in the GN arm (*n* = 23), those who had undergone surgery had higher scores than those who had not (*n* = 10) (mASS-14 = 2.70 vs. 5.96, Adj-*p* = 0.1769; BAI = 6 vs. 2.3, Adj-*p* = 0.248). Among individuals without prior surgical experience (*n* = 21), controls (*n* = 11) had higher scores than GN participants (*n* = 10) (mASS-14 = 6.0 vs. 2.7, Adj-*p* = 0.281; BAI = 6.73 vs. 2.3, Adj-*p* = 0.218). Controls with surgical history had higher scores than GN participants without surgical history (BAI = 7.52 vs. 2.3, Adj-*p* = 0.048; mASS-14 = 6.33 vs. 2.7, Adj-*p* = 0.114) (Figure S6). Among individuals on chronic medication (*n* = 29), the GN group showed lower scores (*n* = 12, BAI = 3.58; mASS-14 = 3.42) compared to controls (*n* = 17, BAI = 9.0, Adj-*p* = 0.0294; mASS-14 = 7.29, Adj-*p* = 0.069) (Figure S7). Among patients under surveillance (*n* = 4), GN participants had lower scores (*n* = 2, BAI = 0; mASS-14 = 1.0) compared to controls (*n* = 2, BAI = 7.5, Adj-*p* = 0.70; mASS-14 = 7.5, Adj-*p* = 0.62) (Figure S8). Among patients with solid lesions (*n* = 44), GN participants had lower BAI scores (*n* = 19, BAI = 4.84) compared to controls (*n* = 25, BAI = 7.76, *p* = 0.0712) (Figure S9). Among patients with prior hospital admissions (*n* = 51), the GN group (*n* = 26) showed lower scores compared to controls (*n* = 25) (BAI = 5.12 vs. 7.76, Adj-*p* = 0.292; mASS-14 = 5.46 vs. 6.4, Adj-*p* = 0.865) (Figure S10). Patients with suspected metastases (*n* = 8) in the GN group (*n* = 3) showed a tendency toward lower BAI scores (mean 3.33 [±2.89]) compared to the control group (mean 14.8 [±14.3], Adj-*p* = 0.007), and similarly mASS-14 score (mean 3.0 [±1.73] for GN vs. mean 8.20 [±9.07] for controls, Adj-*p* = 0.348) (Figure S12). The different cut-off of intercurrent time did not reveal any differences. MASS-14 showed *p*-values of 0.42, 0.054, and 0.47 at the 30-, 60-, and 90-day cut-offs, respectively. Similarly, BAI showed *p*-values of 0.42, 0.30, and 0.60 at the same time points (Table S1).

## 4. Discussion

The utilization of graphic narratives in the medical field, though not widespread, is gaining recognition for its potential in patient care management across various pathological conditions. A recent study screened 125 academic health science libraries in America to identify graphic medicine titles among their collections. Only 14% of them did not have any graphic medicine titles available, suggesting that academic health science libraries are interested in graphic medicine [33]. The American Cancer Society has pioneered this approach in cancer prevention, employing a comic book to address the barriers to cervical cancer screening [15]. In the sensitive domain of AIDS, comic books have contributed positively to the discourse surrounding this infectious and severe condition [16]. Graphic novels have also been used as “awareness comics” thanks to their potential to improve acquired knowledge and behaviour changes in populations at risk of developing diseases, such as patients with metabolic risk factors who could develop liver diseases [34]. Another significant role of graphic novels is their impact on stress and anxiety management in patients awaiting biopsy for potentially malignant disorders. In an RCT, graphic novels significantly improved the ability of patients to tolerate anxiety while waiting for an oral biopsy in suspected oral cancers [18]. Nowadays, patients can experience intense and immediate fear and/or anxiety prior to endoscopy and/or pancreatic biopsy, and this discomfort typically manifests in the waiting room, possibly leading to negative intrusive thoughts (e.g., “It will hurt.” or “I will be diagnosed with a terrible disease.”) [35]. This pilot study is a hypothesis-generating study for investigating the feasibility of a graphic novel intervention in reducing anxiety among patients awaiting EUS-FNB for pancreatic lesions. The primary anxiety exploratory measure was the BAI, a validated instrument, whereas the modified DASS-21 (mASS-14) was used as an exploratory/secondary measure capturing both anxiety and stress dimensions. The overall mean anxiety scores for both arms were relatively low (mean BAI and mASS-14 4.97 and 4.88 for graphic, 6.22 and 7.25 for controls), suggesting that the general anxiety levels in this patient cohort might not be exceptionally high, confirmed by the low prevalence of anxiety (4.6% according to BAI and 13.8% according to mASS-14). The observed trends in the graphic novel group (lower mean BAI and mASS-14 scores compared to controls) suggest a slight influence that warrants further investigation, even though *p*-values are only indicative in a pilot study, while mean values are far more important [36]. Interestingly, the graphic novel group showed a lower mean BAI compared to controls among patients with children (4.66 vs. 7.63), pointing to individuals balancing medical concerns with family responsibilities. However, controls with surgical history had higher scores than graphic novel participants without surgical history (BAI = 7.52 vs. 2.3; mASS-14 = 6.33 vs. 2.7), further highlighting the need to explore the potential benefit of GN for those new to such procedures. The graphic novel appears to give different results in patients on chronic medication assumption or those enrolled in surveillance programs, as they reported lower anxiety scores compared to controls, indicating a potential role in managing anxiety in individuals dealing with ongoing health management or uncertainty. Moreover, patients with solid lesions and, notably, those with suspected metastases in the graphic novel group also showed a tendency towards lower BAI and mASS-14 scores.

This study has the strength of being a pilot study representing a novel application of graphic novels in endoscopy. Another significant strength lies in the identification of specific patient populations in which the graphic novel showed potential benefits in anxiety reduction, which need to be further investigated. The easy application and use of this tool indicate the practical feasibility and acceptability of integrating it into clinical practice without causing additional discomfort to patients. Moreover, the graphic novel itself was designed with careful consideration of the patient experience, giving the ability to communicate effectively and provide comfort. Despite its strengths, the study also presents several limitations to acknowledge when interpreting its findings. Firstly, it is a preliminary study with the aim of being a hypothesis-generating study, and, furthermore, the mean anxiety scores in both groups were relatively low. This might have created a floor effect, making it difficult to detect a significant reduction in anxiety even if the graphic novel had a slight positive impact. Additionally, the relatively small sample size limits both statistical power and generalizability, as is typical in pilot studies, where effect size estimates and *p*-values are highly unstable. Consequently, the findings placed greater emphasis on descriptive statistics, particularly mean values and confidence intervals, rather than on statistical significance, and needed caution when interpreting differences within small subgroups [36]. Another limitation of this pilot study is the baseline imbalance in clinical presentation and the prevalence of cancer, with the latter being higher in the control group. The SMDs for clinical presentation and histological diagnosis are moderate to large (around −0.54 and −0.41), reflecting an important imbalance in symptom status and final diagnosis that could confound anxiety outcomes and must be considered when interpreting these results. Therefore, the collection of baseline anxiety measures, use of an attention-matched control condition, and stratified randomization by key prognostic factors (such as clinical presentation) should be recommended in future trials. Given the small sample size and the lack of stratification by final diagnosis, the imbalance in clinical presentation and pancreatic cancer between groups is most likely due to chance rather than to a failure of the randomization process. Another limitation is that post-procedural BAI and mASS-14 scores may be influenced by sedation or anaesthesia, procedural relief, and the overall experience, and therefore do not represent a specific measure of waiting-room anxiety but rather an exploratory picture of peri-procedural anxiety. Future studies should include repeated assessments to better isolate the intervention’s impact on anticipatory anxiety. A further limitation is the absence of blinding due to the nature of the intervention, as mentioned above, which may have introduced measurement bias. However, the study is single-center and focuses specifically on patients undergoing EUS-FNB for pancreatic lesions, which may further limit the generalizability to diverse healthcare settings and may not be directly transferable to other medical procedures or patient populations. Moreover, the transferability of our findings to centres routinely performing EUS-FNB under conscious sedation is limited. Therefore, these results are directly applicable mainly to centres using general anaesthesia or deep sedation for EUS-FNB, so the graphic novels should be viewed as a communication tool potentially adaptable to settings with conscious sedation, but needing specific validation. Another limitation is that the graphic novel arm is inherently involved in additional ‘extra attention’, as patients received not only standard counselling and extra material, but also further interaction. As a result, part of the observed anxiety reduction may reflect increased time and attention rather than a specific effect of the graphic format itself.

## 5. Conclusions

This exploratory, hypothesis-generating pilot study showed a slight, non-significant trend toward a reduction in anxiety levels with the use of a graphic novel intervention among patients undergoing EUS-FNB for pancreatic lesions, compared with standard care (BAI 4.88 ± 3.24 vs. 7.25 ± 7.11). These preliminary results warrant further investigation in larger, adequately powered studies, providing a basis for future hypotheses and more targeted investigations in managing patient anxiety during diagnostic procedures. In the near future, clinical trials will be needed to evaluate graphic novels in the management of anxiety and stress tolerance.

## Figures and Tables

**Table 1:** Characteristics of patients (*n* = 65).

Characteristics of Patients at Baseline, *n* (%) or Mean (±SD)		Randomization			
	Overall, *n* = 65	Graphic Novel, *n* = 33	Controls, *n* = 32	*p*-Value *	SMD
Age, years	64.5 (13.6)	64.9 (13.9)	64 (13.5)	0.804	0.06176
Male (%)	36 (55.4)	20 (60.6)	16 (50)	0.459	−0.2112
BMI, Kg/m^2^	26.6 (4.6)	26.9 (4.8)	26.3 (4.4)	0.510	0.16443
Smoker				0.823	0.0362
Active	21 (32.3)	10 (47.6)	11 (52.4)		
Previous	3 (4.6)	2 (66.7)	1 (33.3)		
No	41 (63.1)	21 (51.2)	20 (48.8)		
Diabetes	24 (36.9)	9 (37.5)	15 (62.5)	0.127	−0.4080
Family history pancreatic cancer	9 (13.9)	5 (55.6)	4 (44.4)	0.757	0.0756
Degree of blood ties				0.764	
1st-degree relatives	5 (55.6)	3 (60)	2 (40)		
2nd-degree relatives	4 (44.4)	2 (50)	2 (50)		
Charlson-Comorbidity Index	3.4 (1.6)	3.36 (1.62)	3.48 (1.50)	0.759	−0.0770
Educational level				0.872	−0.0499
Elementary school	11 (16.9)	5 (45.5)	6 (54.5)		
Middle school	21 (32.3)	12 (57.1)	9 (42.9)		
High school	28 (43.1)	14 (50)	14 (50)		
University degree	5 (7.7)	2 (40)	3 (60)		
Previous hospital admission	51 (78.5)	26 (51)	25 (49)	0.948	0.0159
Type of previous hospital admission				0.914	−0.0670
Outpatient	2 (3.9)	1 (50)	1 (50)		
Day Hospital	5 (9.8)	3 (60)	2 (40)		
Inpatient	44 (86.3)	22 (50)	22 (50)		
Previous surgery	44 (67.7)	23 (52.3)	21 (47.7)	0.726	0.0858
Type of surgery				0.318	−0.2958
Major	38 (86.4)	21 (55.3)	17 (44.7)		
Minor	6 (13.6)	2 (33.3)	4 (66.7)		
Surgical adverse events (AEs)	0 (0)	-	-	-	-
Chronic drug intake (yes)	29 (44.6)	12 (41.4)	17 (58.6)	0.174	−0.3367

* Fisher’s exact test or ꭓ^2^ test. SD: standard deviation; BMI: body mass index; SMD: standardized mean differences (SMD).

**Table 2:** Characteristics of the pancreatic lesions, including histology after endoscopic ultrasound with fine needle biopsy.

	Overall, *n* = 65	Graphic Novel, *n* = 33	Control, *n* = 32	*p* Value *	SMD
Pancreatic lesion size, mm, mean (±SD)	34.9 (19.4)	35 (22.3)	34.8 (16.3)	0.961	0.0121
Ca 19.9, U/mL, mean (±SD)	688 (2446.4)	553.3 (2608.4)	826.5 (2300.7)	0.656	−0.1111
Type of lesion, *n* (%)				0.195	−0.1603
Cystic	17 (26.2)	11 (64.7)	6 (35.3)		
Solid	44 (67.7)	19 (43.2)	25 (56.8)		
Mixed	4 (6.3)	3 (75)	1 (25)		
Clinical presentation, *n* (%)				0.014	−0.5419
Incidental	35 (53.9)	23 (65.7)	12 (34.3)		
Symptomatic	26 (40)	8 (30.8)	18 (69.2)		
Surveillance	4 (6.2)	2 (50)	2 (50)		
Histological diagnosis, *n* (%)				0.003	−0.4146
Non diagnostic	6 (9.23)	4	2		
Hepatocellular carcinoma	1 (1.54)	1	0		
Mesenterial Cyst	1 (1.54)	0	1		
Serous cystoadenoma	4 (6.15)	1	3		
Gastrointestinal tumor (GIST)	1 (1.54)	1	0		
IPMN without dysplasia/atypical cells	2 (3.1)	2	0		
Accessory spleen	2 (3.1)	1	1		
Negative for neoplasia	11 (16.9)	9	2		
Neuroendocrine tumor	8 (12.3)	5	3		
Chronic pancreatitis mass forming	1 (1.54)	1	0		
Pancreatic cancer (PDAC)	26 (40)	6	20		
Peripancreatic collections	2 (3.1)	2	0		

* Fisher’s exact test or ꭓ^2^ test. PDAC: pancreatic ductal adenocarcinoma; SMD: standardized mean differences (SMD).

**Table 3:** Comparison between interventional group (Graphic Novel) and control group.

Scale	Overall, *n* = 65	Graphic Novel, *n* = 33	Controls, *n* = 32	*p* Value *
**BAI, mean (±SD)**	6.05 (±5.58)	4.88 (3.24)	7.25 (7.11)	0.092
**BAI grading, *n* (%)**				0.203
Minimal	41 (63.1)	24 (58.5)	17 (41.5)	
Mild	21 (32.3)	9 (42.9)	12 (57.1)	
Moderate	1 (1.5)	0 (0)	2 (100)	
Severe	2 (3.1)	0	1 (100)	
**mASS-14, mean (±SD)**	5.58 (±4.45)	4.97 (3.7)	6.22 (5.1)	0.261
**Intercurrent time, mean (±SD), days**	36.3 (35.2)			

* Fisher’s exact test or ꭓ^2^ test. SD: standard deviation; BAI: Beck Anxiety Inventory; mASS-14: modified Anxiety Stress Scales-21.

## Data Availability

The data presented in this study are available on request from the corresponding author. The data are not publicly available due to the internal policy for sharing the dataset.

## Supplementary Materials

The following supporting information can be downloaded at: https://www.mdpi.com/article/10.3390/healthcare14060699/s1, Table S1: Differences in mASS-14 and BAI at different cut-off of intercurrent time (30, 60 and 90 days); Figure S1. Beck Anxiety Inventory (BAI); Figure S2: The modified version of DASS-21, named mASS-14; Figure S3: Overview of study protocol. EUS-FNB = endoscopic ultrasound with fine needle biopsy; Figure S4. Interaction of intervention (Graphic novel) on BAI score (A) and mASS-14 score (B) based on degree of familiarity; Figure S5: Interaction of intervention (Graphic novel) on mASS-14 (A) and BAI (B) score based on family support (children yes/no); Figure S6: Interaction of intervention (Graphic novel) on BAI (A) o mASS-14 (B) score based on previous surgery (yes/no); Figure S7: Interaction of intervention (Graphic novel) on BAI (A) and mASS-14 (B) score based on chronic drug administration (yes/no); Figure S8: Interaction of intervention (Graphic novel) on BAI (A) and mASS-14 (B) score based on clinical presentation (Incidental/Symptomatic/Surveillance); Figure S9: Interaction of intervention (Graphic novel) on BAI score (A) and mASS-14 (B) based on type of lesion (cystic/solid/cystic-solid); Figure S10: Interaction of intervention (Graphic novel) on BAI score (A) and mASS-14 (B) based on previous hospital admission; Figure S11: Interaction of intervention (Graphic novel) on BAI score (A) and mASS-14 (B) based on level of education; Figure S12: Interaction of intervention (Graphic novel) on BAI score (A) and mASS-14 (B) based on suspected metastasis; CONSORT 2010 checklist of information to include when reporting a pilot or feasibility trial; The TIDieR (Template for Intervention Description and Replication) Checklist.

## Abbreviations

EUS	Endoscopic ultrasound
EUS-FNB	EUS with fine needle biopsy
BAI	Beck Anxiety Inventory
mDASS-21	modified version of the Depression Anxiety Stress Scales-21
mASS-14	modified Anxiety Stress Scales-14
eCRF	electronic case report form
GLMs	generalized linear models
AIDS	Acquired Immunodeficiency Syndrome

## References

[1] Siegel R.L., Kratzer T.B., Wagle N.S., Sung H., Jemal A. (2026). Cancer Statistics, 2026. CA Cancer J. Clin..

[2] Kawaguchi K., Maeda M., Murata F., Endo M., Nakashima Y., Fukuda H. (2026). Incidence and Temporal Trends of Depression and Anxiety Following Cancer Diagnosis in Japan: Insights from the LIFE Study. Int. J. Cancer.

[3] Clark D.A., Beck A.T. (2010). Cognitive Theory and Therapy of Anxiety and Depression: Convergence with Neurobiological Findings. Trends Cogn. Sci..

[4] Semenenko E., Banerjee S., Olver I., Ashinze P. (2023). Review of Psychological Interventions in Patients with Cancer. Support. Care Cancer.

[5] Green M.J., Myers K.R. (2010). Graphic Medicine: Use of Comics in Medical Education and Patient Care. BMJ.

[6] Nickerson S.M. (2018). Seeking Graphic Medicine Narratives. CMAJ Can. Med. Assoc. J..

[7] De Stefano A., Rusciano I., Moretti V., Scavarda A., Green M.J., Wall S., Ratti S. (2023). Graphic Medicine Meets Human Anatomy: The Potential Role of Comics in Raising Whole Body Donation Awareness in Italy and beyond. A Pilot Study. Anat. Sci. Educ..

[8] Magnan R.E., Hamilton W.K., Shorey-Fennell B., Cameron L.D. (2021). Experimental Test of the Educational Impact of the Newly Proposed FDA Graphic Cigarette Warnings Among U.S. Adults. Ann. Behav. Med..

[9] Merks P., Cameron J., Bilmin K., Świeczkowski D., Chmielewska-Ignatowicz T., Harężlak T., Białoszewska K., Sola K.F., Jaguszewski M.J., Vaillancourt R. (2021). Medication Adherence and the Role of Pictograms in Medication Counselling of Chronic Patients: A Review. Front. Pharmacol..

[10] Williams L., Harstäde C.W., Anderson N., Deshmukh A., Gayton A., Gott M., Guo P., Nicol J., Tavares T., Waterworth S. (2024). Nursing Students’ Reactions to a Graphic Novel: A Multi-National Descriptive Qualitative Study. Nurse Educ. Today.

[11] Lesińska-Sawicka M. (2023). Using Graphic Medicine in Teaching Multicultural Nursing: A Quasi-Experimental Study. BMC Med. Educ..

[12] Venkatesan S., Murali C. (2022). Graphic Medicine and the Critique of Contemporary U.S. Healthcare. J. Med. Humanit..

[13] Al-Jawad M. (2015). Comics Are Research: Graphic Narratives as a New Way of Seeing Clinical Practice. J. Med. Humanit..

[14] Branscum P., Sharma M., Wang L.L., Wilson B., Rojas-Guyler L. (2013). A Process Evaluation of a Social Cognitive Theory-Based Childhood Obesity Prevention Intervention: The Comics for Health Program. Health Promot. Pract..

[15] Krakow M. (2017). Graphic Narratives and Cancer Prevention: A Case Study of an American Cancer Society Comic Book. Health Commun..

[16] Czerwiec M.K. (2018). Representing AIDS in Comics. AMA J. Ethics.

[17] Venkatesan S., Peter A.M. (2020). Feminine Famishment: Graphic Medicine and Anorexia Nervosa. Health.

[18] Bazzano M., Mauceri R., Marcon G., Campisi G. (2023). Reducing the Anxiety of Patients Undergoing an Oral Biopsy by Means of Graphic Novels: An Open-Label Randomized Clinical Trial. Med. Oral Patol. Oral Cirugia Bucal.

[19] Eldridge S.M., Chan C.L., Campbell M.J., Bond C.M., Hopewell S., Thabane L., Lancaster G.A., PAFS consensus group (2016). CONSORT 2010 Statement: Extension to Randomised Pilot and Feasibility Trials. BMJ.

[20] Hoffmann T.C., Glasziou P.P., Boutron I., Milne R., Perera R., Moher D., Altman D.G., Barbour V., Macdonald H., Johnston M. (2014). Better Reporting of Interventions: Template for Intervention Description and Replication (TIDieR) Checklist and Guide. BMJ.

[21] Beck A.T., Epstein N., Brown G., Steer R.A. (1988). An Inventory for Measuring Clinical Anxiety: Psychometric Properties. J. Consult. Clin. Psychol..

[22] Ng F., Trauer T., Dodd S., Callaly T., Campbell S., Berk M. (2007). The Validity of the 21-Item Version of the Depression Anxiety Stress Scales as a Routine Clinical Outcome Measure. Acta Neuropsychiatr..

[23] Rizzo G.E.M., Traina M., Tarantino I. (2024). The Impact of a Graphic Novel on Anxiety and Stress in Patients Undergoing Endoscopic Ultrasound with Fine Needle Biopsy for Pancreatic Lesions: A Pilot Study Protocol. Front. Gastroenterol..

[24] Sica C., Ghisi M., Lange M.A. (2007). The Italian versions of the Beck Anxiety Inventory and the Beck Depression In-ventory-II: Psychometric properties and discriminant power. Leading-Edge Psychological Tests and Testing Research.

[25] Lovibond P.F., Lovibond S.H. (1995). The structure of negative emotional states: Comparison of the Depression Anxiety Stress Scales (DASS) with the Beck Depression and Anxiety Inventories. Behav. Res. Ther..

[26] Bottesi G., Ghisi M., Altoè G., Conforti E., Melli G., Sica C. (2015). The Italian version of the Depression Anxiety Stress Scales-21: Factor structure and psychometric properties on community and clinical samples. Compr Psychiatry..

[27] Brown T.A., Chorpita B.F., Korotitsch W., Barlow D.H. (1997). Psychometric Properties of the Depression Anxiety Stress Scales (DASS) in Clinical Samples. Behav. Res. Ther..

[28] Soria-Reyes L.M., Alarcón R., Cerezo M.V., Blanca M.J. (2024). Psychometric Properties of the Depression Anxiety Stress Scales (DASS-21) in Women with Breast Cancer. Sci. Rep..

[29] Pezirkianidis C., Karakasidou E., Lakioti A., Stalikas A., Galanakis M. (2018). Psychometric Properties of the Depression, Anxiety, Stress Scales-21 (DASS-21) in a Greek Sample. Psychology.

[30] Thapa D.K., Visentin D., Kornhaber R., Cleary M. (2022). Psychometric Properties of the Nepali Language Version of the Depression Anxiety Stress Scales (DASS-21). Nurs. Open.

[31] Bados A., Solanas A., Andrés R. (2005). Psychometric Properties of the Spanish Version of Depression, Anxiety and Stress Scales (DASS). Psicothema.

[32] Austin P.C. (2009). Balance Diagnostics for Comparing the Distribution of Baseline Covariates between Treatment Groups in Propensity-Score Matched Samples. Stat. Med..

[33] Chan J., Berg M.H., Bullers K., Lue T.Y. (2025). Graphic Medicine in Academic Health Science Library Collections. J. Med. Libr. Assoc. JMLA.

[34] Alemany-Pagès M., Tavares R., Azul A.M., Ramalho-Santos J. (2024). A Healthy Liver Will Always Deliver: Impact Study of a Non-Alcoholic Fatty Liver Disease (NAFLD) Awareness Comic. Health Commun..

[35] Beck A.T., Rush A.J. (1985). A Cognitive Model of Anxiety Formation and Anxiety Resolution. Issues Ment. Health Nurs..

[36] Leon A.C., Davis L.L., Kraemer H.C. (2011). The Role and Interpretation of Pilot Studies in Clinical Research. J. Psychiatr. Res..

