# Supplementary material for: Graphic Novel for Patients Affected by Pancreatic Lesions Undergoing Endoscopic Ultrasound with Fine Needle Biopsy: A Pilot Randomized Study
Source: Healthcare (Basel). 2026 Mar 10;14(6):699. doi: 10.3390/healthcare14060699 (PMC13025995; doi:10.3390/healthcare14060699)
Supplement: Supplementary file 1 [file healthcare-14-00699-s001.zip › healthcare-4144697-supplementary.pdf]

Supplementary Materials

Assessing Anxiety

Anxiety and stress were assessed using two instruments: the Beck Anxiety Inventory (BAI) and a modified version of the Depression Anxiety Stress Scales-21 (mDASS-21, referred to as mASS-14).

Beck Anxiety Inventory (BAI)

The BAI is a 21-item self-report measure of anxiety severity developed by Beck et al. [1]. The original validation study established excellent psychometric properties in psychiatric outpatients, with a Cronbach's alpha of 0.92. The inventory focuses on somatic and subjective symptoms of anxiety rated on a 4-point scale (0 = Not at all to 3 = Severely). Total scores range from 0 to 63, with established cutoffs: 0–7 (minimal), 8–15 (mild), 16–25 (moderate), and 26–63 (severe) anxiety. The Italian version was validated by Sica & Ghisi (2007, The Italian Versions of the Beck Anxiety Inventory and the Beck Depression Inventory-II: Psychometric Properties and Discriminant Power), demonstrating good to excellent internal consistency ([Cronbach Index] $\alpha = 0.87–0.90$) and strong discriminant validity in non-clinical samples (N = 1,485) and anxious patients. In our sample, the BAI showed excellent internal consistency ($\alpha = 0.858$).

Modified Depression Anxiety Stress Scales (mDASS-21/mASS-14)

The mASS-14 comprises the anxiety and stress subscales (14 items) from the DASS-21, excluding the depression subscale. The original 42-item DASS was developed by Lovibond & Lovibond [2] to maximize discrimination between depression and anxiety symptoms. The 21-item short form was validated by Ng et al. [3] in 786 psychiatric inpatients, showing significant pre-post treatment sensitivity and convergent validity with clinical measures. Brown et al. [4] confirmed excellent internal consistency (anxiety $\alpha = 0.89$, stress $\alpha = 0.93$) and a stable three-factor structure in clinical samples (N = 437).

We excluded the depression subscale because: (a) depression was not relevant to the acute pre-procedural anxiety state being assessed, and (b) our focus was specifically on anxiety (physiological arousal, situational fear) and stress (tension, irritability, agitation) dimensions pertinent to the immediate medical context. Therefore, the anxiety and stress subscales maintain their psychometric properties when administered separately. Bottesi et al. [5] demonstrated that the Italian DASS-21 three-factor oblique model showed good fit (CFI = 0.968, RMSEA = 0.046), supporting distinct anxiety and stress factors. Brown et al. [4] reported Cronbach's alphas of 0.89 (anxiety) and 0.93 (stress) that remained stable across diagnostic groups. Precedent for independent subscale use exists in published studies assessing specific symptom domains.

Therefore, the Italian DASS-21 was validated by Bottesi et al. [5] in 417 community participants and 57 clinical patients. The anxiety subscale showed $\alpha = 0.74$ (community) and $\alpha = 0.88$ (clinical), while the stress subscale showed $\alpha = 0.85$ (community) and $\alpha = 0.83$ (clinical). Test-retest reliability over two weeks was $r = 0.64$ for

both subscales. The measure demonstrated convergent validity with BAI, BDI-II, and PSQ-20, and discriminated between non-clinical, depressed, and anxious groups.

Each 7-item subscale is scored 0–3 per item (0–21 total). Severity categories follow Lovibond & Lovibond [2]: normal (0–9), mild (10–13), moderate (14–20), severe (21–27), and extremely severe (28+). In our sample, Cronbach's alpha was acceptable (α = 0.771), in line with the literature. However, when exploring it separately based on the subscale, it was poor (α = 0.514) for anxiety subscale and acceptable (α = 0.709) for stress subscale.

However, the modification from DASS-21 to mASS-14 represents a methodological adaptation which was theory-driven: acute procedural anxiety and stress are phenomenologically distinct from clinical depression, and psychometric evidence supported independent subscale validity.

SUPPLEMENTARY TABLES

Supplementary Table S1. Differences in mASS-14 and BAI at different cut-off of intercurrent time (30, 60 and 90 days)

Intercurrent time	Timing: 30 days, mean ± SD			Timing: 60 days, mean ± SD			Timing: 90 days, mean ± SD		
	≤30	>30	p-value	≤60	>60	p-value	≤90	>90	p-value
**mASS-14**	6 ± 4.02	5.1 ± 4.93	0.42	6.06 ± 4.79	4.13 ± 2.8	0.054	5.69 ± 4.5	4 ± 3.56	0.47
**BAI**	6.57 ± 5.4	5.43 ± 5.8	0.42	6.36 ± 4.62	5.06 ± 3.6	0.30	6.11 ± 5.7	5 ± 3.56	0.70

BAI - Beck Anxiety Inventory				
**Items**	SCORING SYSTEM			
	Not at all	Mildly	Moderately	Severely
**Numbness or tingling**	0	1	2	3
**Feeling Hot**	0	1	2	3
**Wobbliness in legs**	0	1	2	3
**Unable to relax**	0	1	2	3
**Fear of worst happening**	0	1	2	3
**Dizzy or lightheaded**	0	1	2	3
**Heart pounding/racing**	0	1	2	3
**Unsteady**	0	1	2	3
**Terrified or afraid**	0	1	2	3
**Nervous**	0	1	2	3
**Feeling of choking**	0	1	2	3
**Hands trembling**	0	1	2	3
**Shaky / unsteady**	0	1	2	3
**Fear of losing control**	0	1	2	3
**Difficulty in breathing**	0	1	2	3
**Fear of dying**	0	1	2	3
**Scared**	0	1	2	3
**Indigestion**	0	1	2	3
**Faint / lightheaded**	0	1	2	3
**Face flushed**	0	1	2	3
**Hot/cold sweats**	0	1	2	3

Supplementary figure S1. Beck Anxiety Inventory (BAI)

Modified Anxiety Stress Scale (mASS-14)				
Items	SCORING SYSTEM			
	Never	Sometimes	Often	Almost always
I found it hard to wind down	0	1	2	3
I was aware of dryness of my mouth	0	1	2	3
I experienced breathing difficulty (e.g., excessively rapid breathing, breathlessness in the absence of physical exertion)	0	1	2	3
I tended to over-react to situations	0	1	2	3
I experienced trembling (e.g., in the hands)	0	1	2	3
I felt that I was using a lot of nervous energy	0	1	2	3
I was worried about situations in which I might panic and make a fool of myself	0	1	2	3
I found myself getting agitated	0	1	2	3
I found it difficult to relax	0	1	2	3
I was intolerant of anything that kept me from getting on with what I was doing	0	1	2	3
I felt I was close to panic	0	1	2	3
I felt that I was rather touchy	0	1	2	3
I was aware of the action of my heart in the absence of physical exertion (e.g., sense of heart rate increase, heart missing a beat)	0	1	2	3
I felt scared without any good reason	0	1	2	3
Total score				

Supplementary figure S2. The modified version of DASS-21, named mASS-14

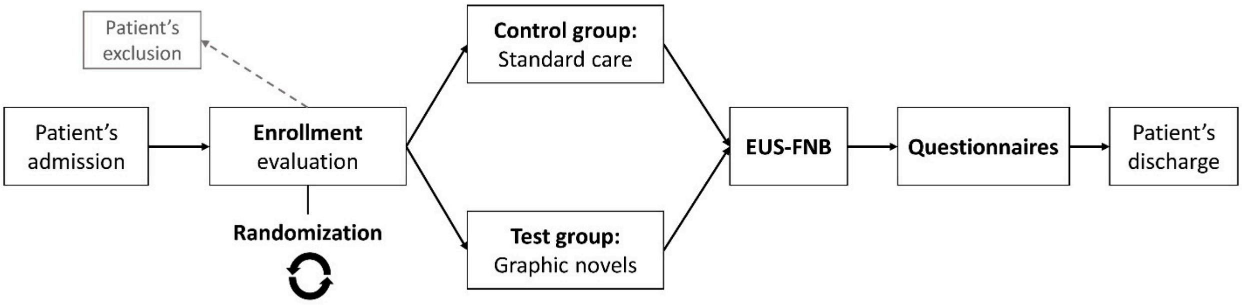

Supplementary figure S3. Overview of study protocol. EUS-FNB = endoscopic ultrasound with fine needle biopsy

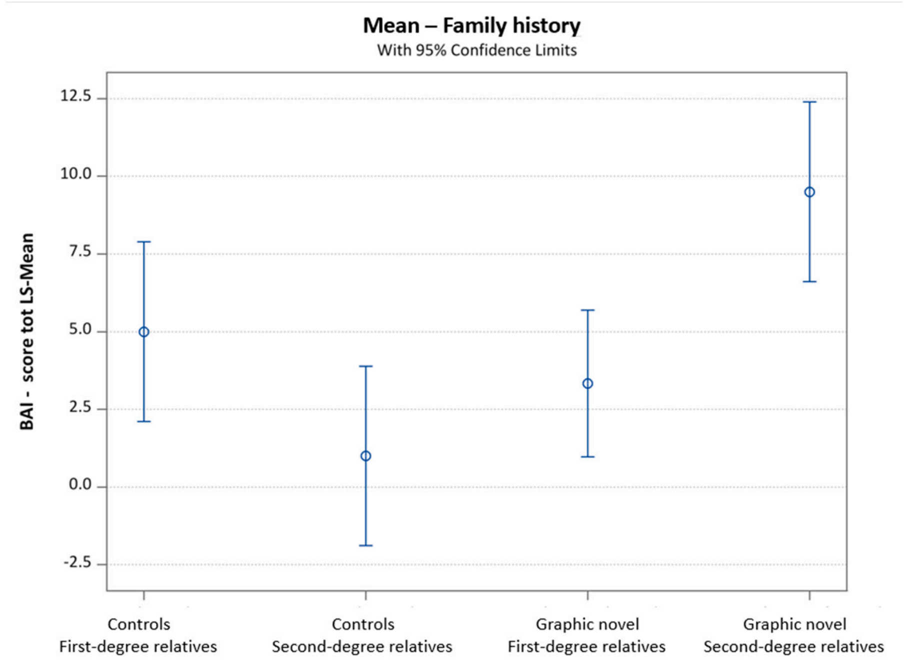

A

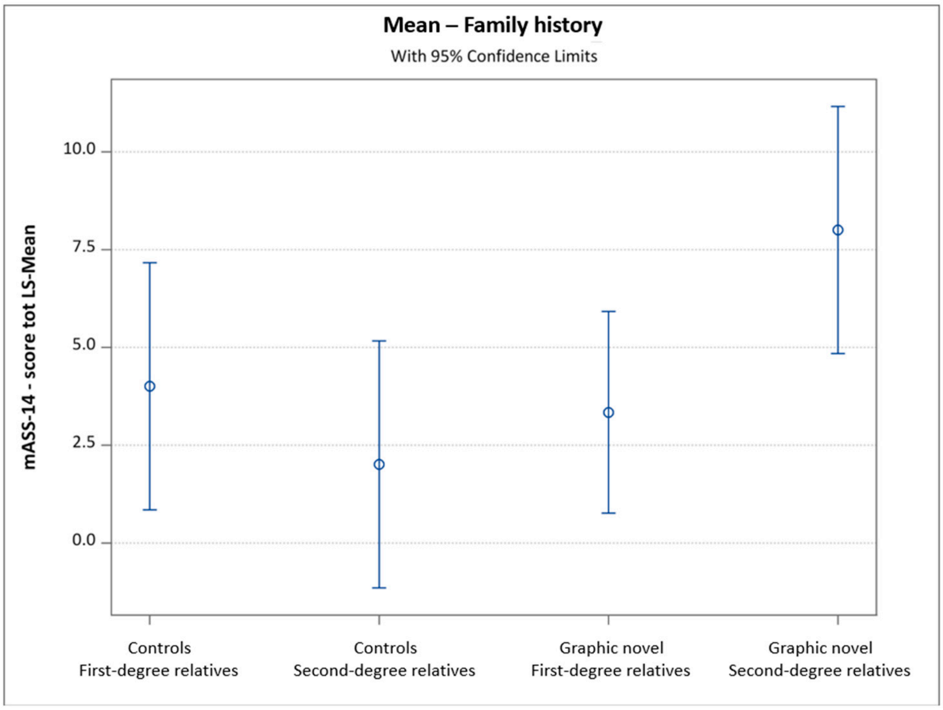

B

Supplementary figure S4. Interaction of intervention (Graphic novel) on BAI score (A) and mASS-14 score (B) based on degree of familiarity.

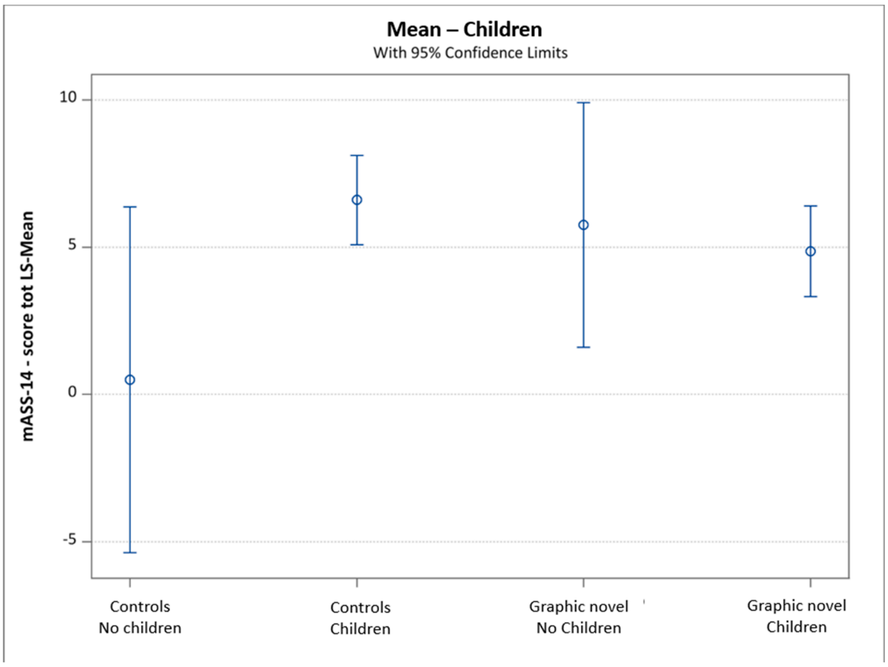

A

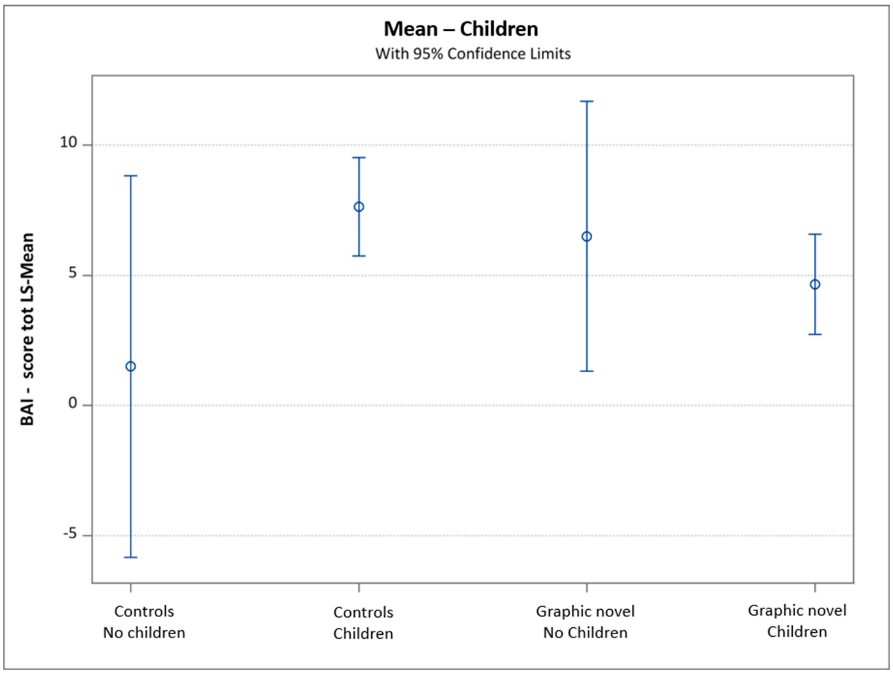

B

Supplementary figure S5. Interaction of intervention (Graphic novel) on mASS-14 (A) and BAI (B) score based on family support (children yes/no).

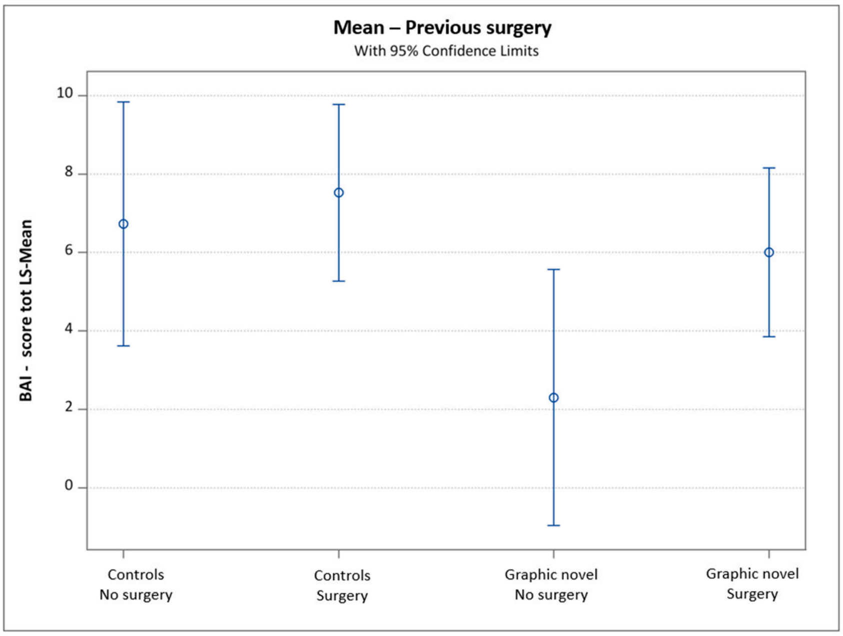

A

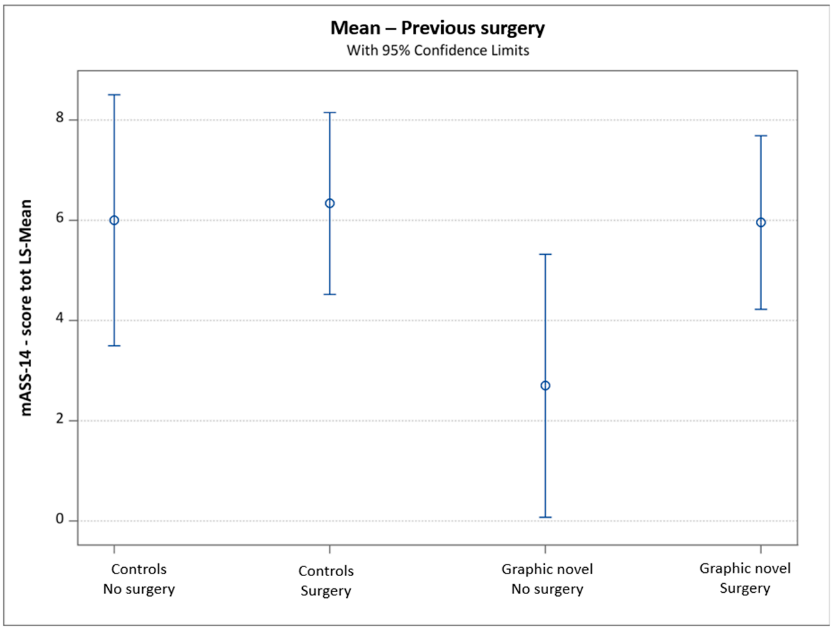

B

Supplementary figure S6. Interaction of intervention (Graphic novel) on BAI (A) o mASS-14 (B) score based on previous surgery (yes/no).

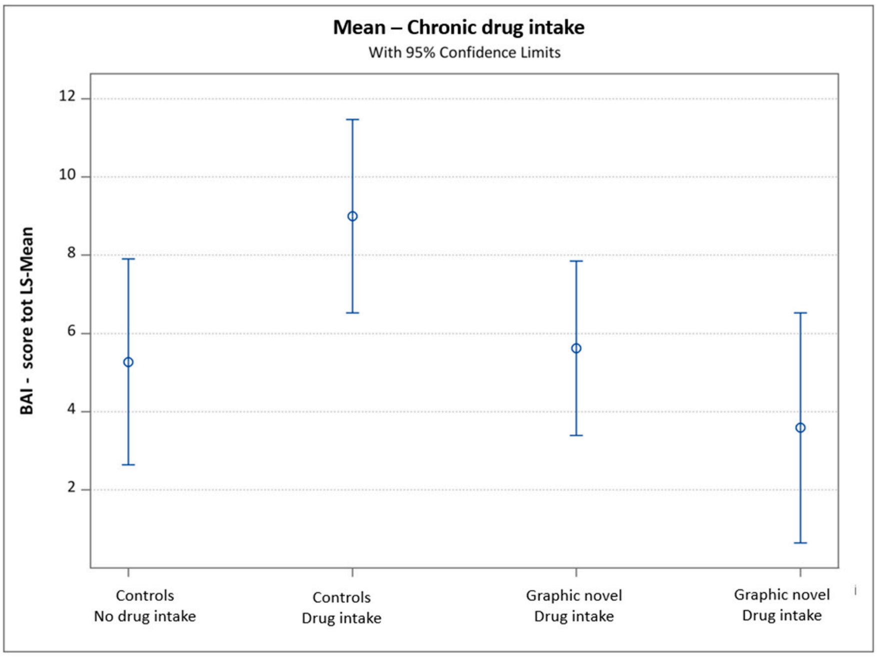

A

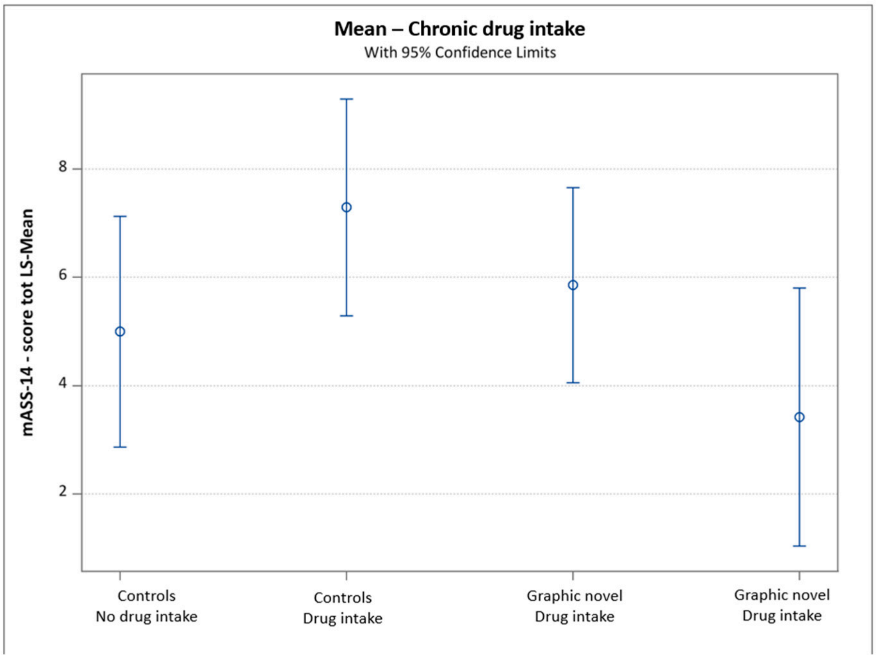

B

Supplementary figure S7. Interaction of intervention (Graphic novel) on BAI (A) and mASS-14 (B) score based on chronic drug administration (yes/no).

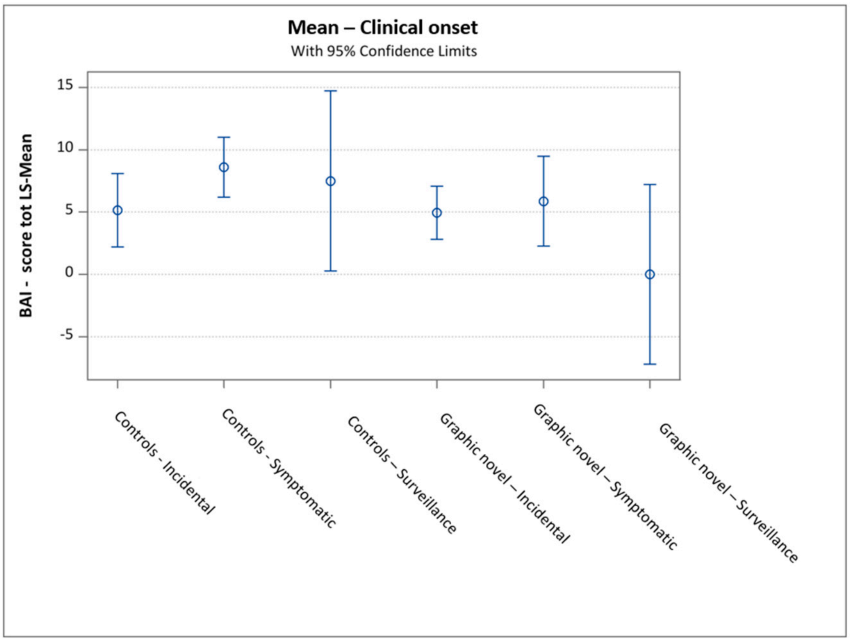

A

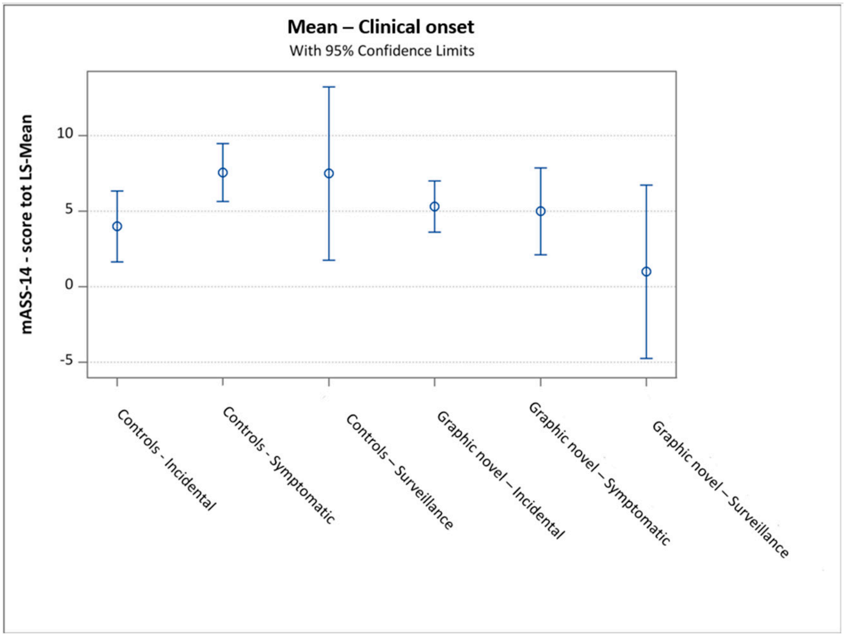

B

Supplementary figure S8. Interaction of intervention (Graphic novel) on BAI (A) and mASS-14 (B) score based on clinical presentation (Incidental/Symptomatic/Surveillance).

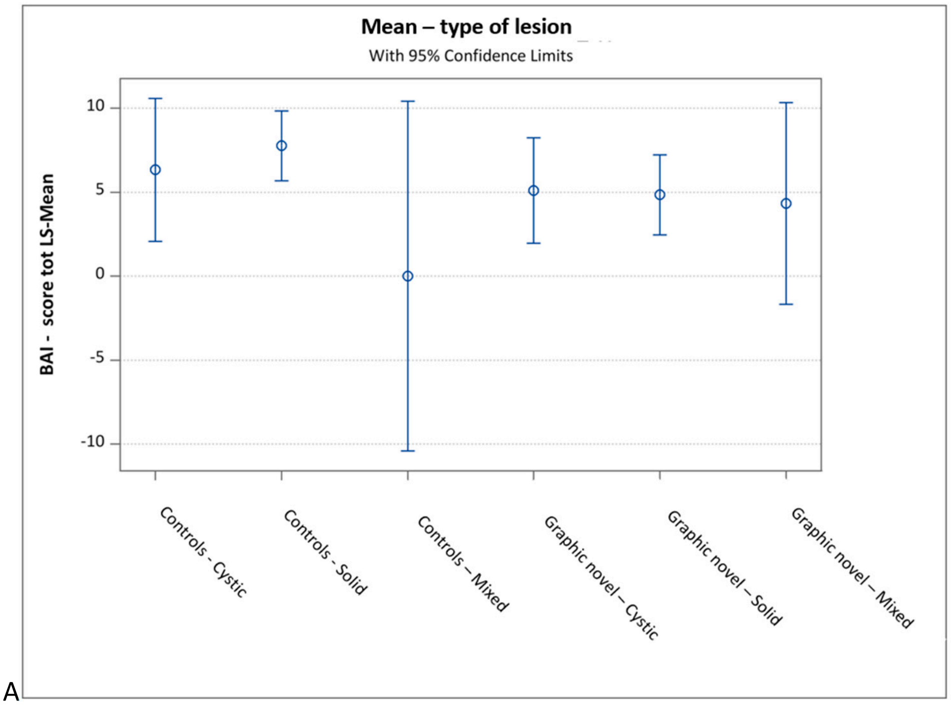

A

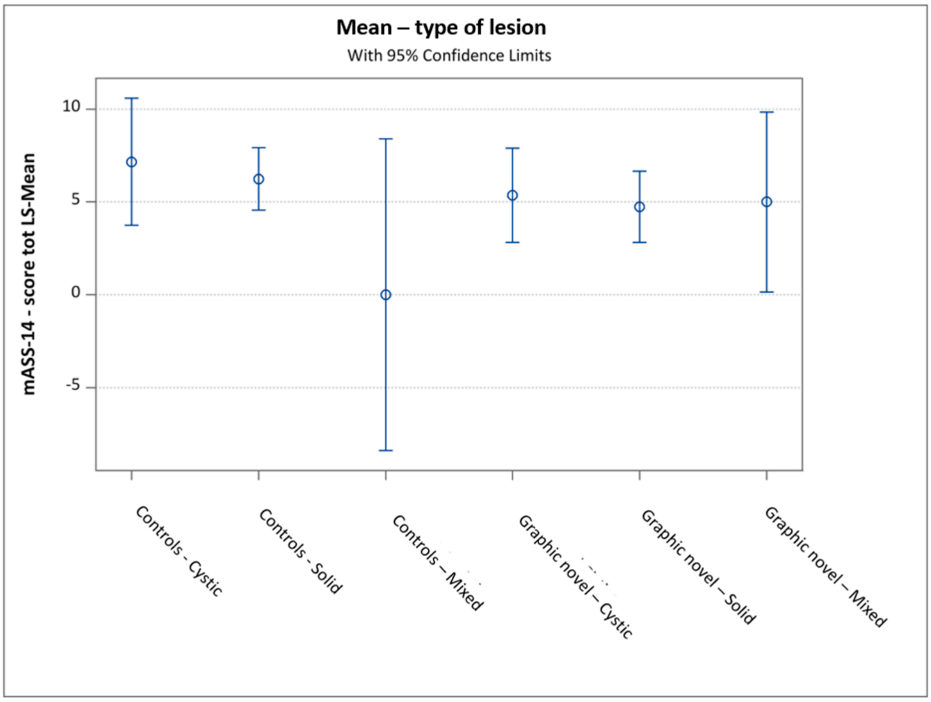

B

Supplementary figure S9. Interaction of intervention (Graphic novel) on BAI score (A) and mASS-14 (B) based on type of lesion (cystic/solid/cystic-solid).

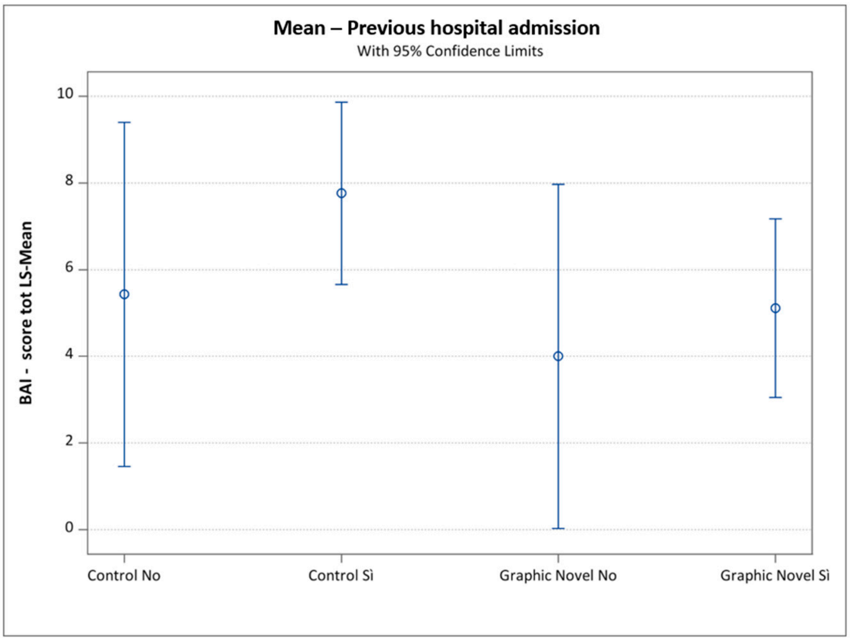

A

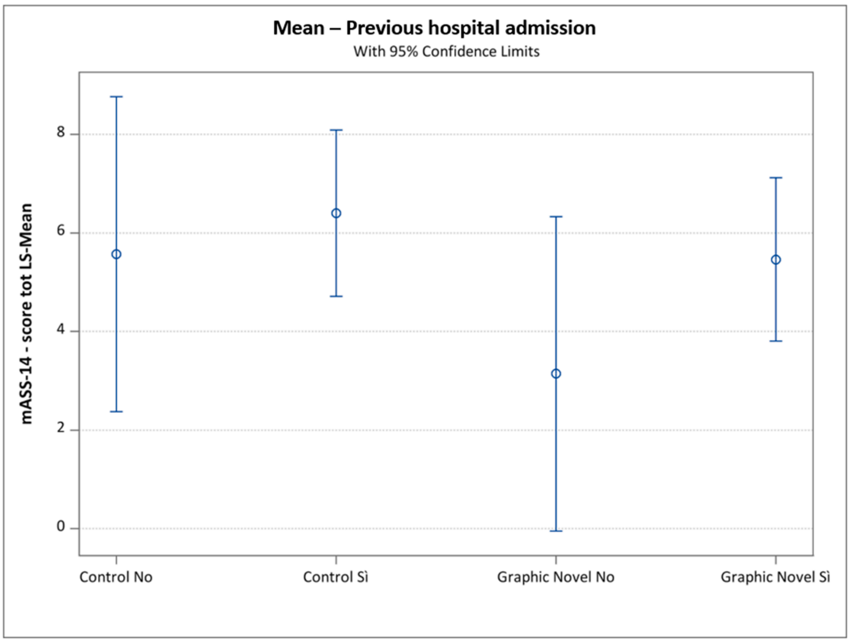

B

Supplementary figure S10. Interaction of intervention (Graphic novel) on BAI score (A) and mASS-14 (B) based on previous hospital admission.

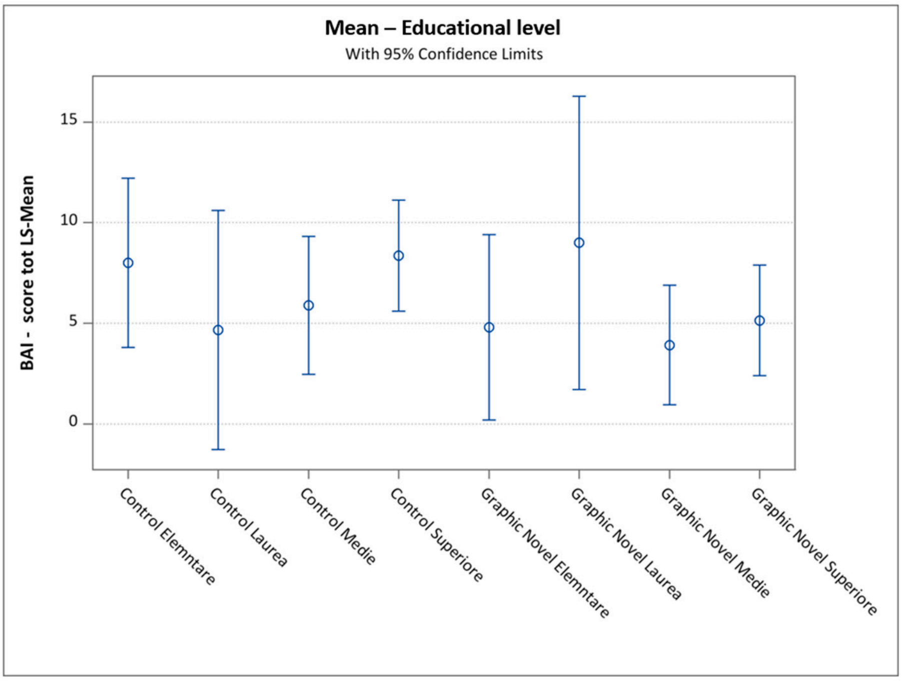

A

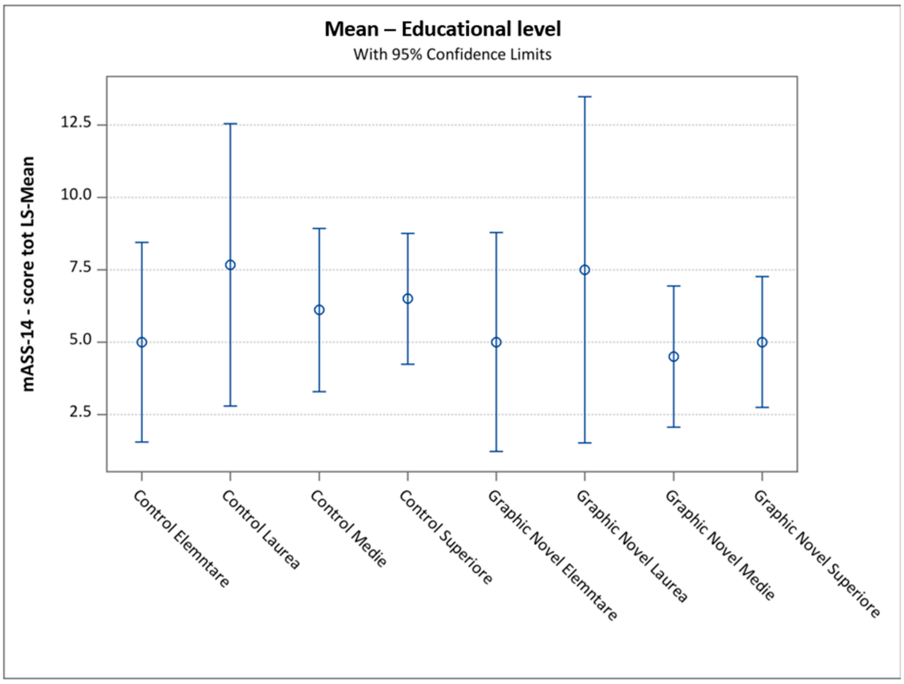

B

Supplementary figure S11. Interaction of intervention (Graphic novel) on BAI score (A) and mASS-14 (B) based on level of education.

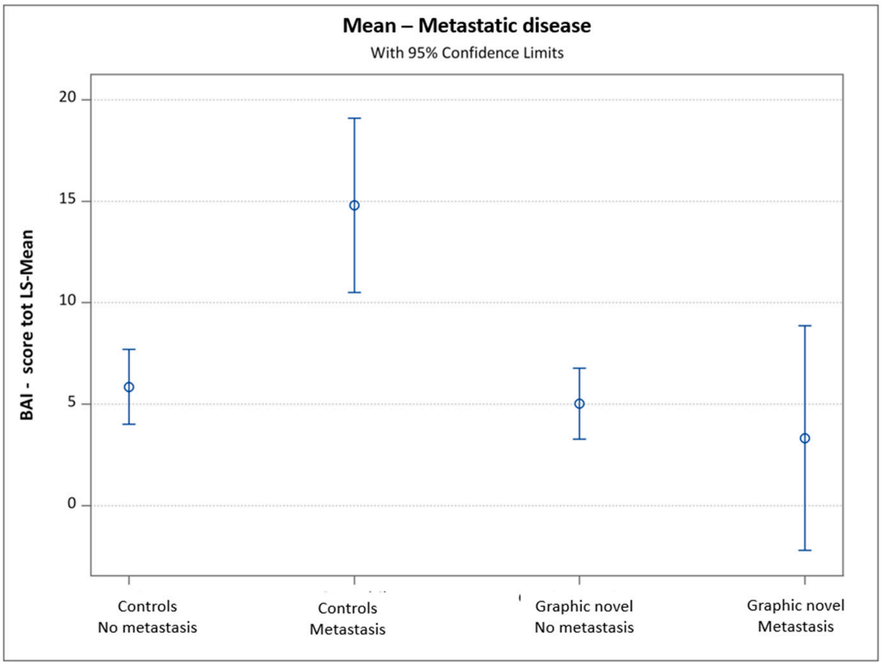

A

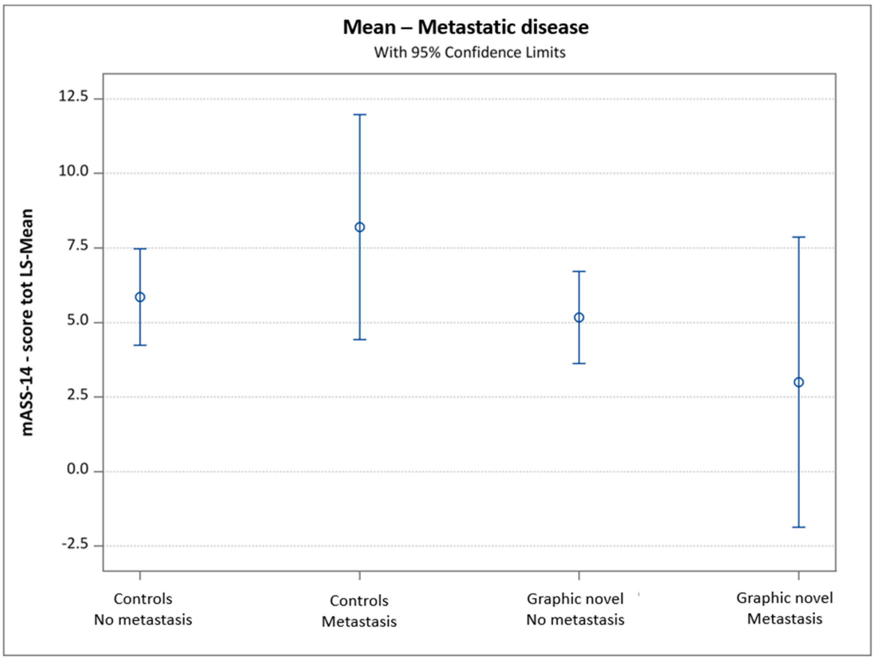

B

Supplementary figure S12. Interaction of intervention (Graphic novel) on BAI score (A) and mASS-14 (B) based on suspected metastasis.

CONSORT 2010 checklist of information to include when reporting a pilot or feasibility trial

Section/Topic	Item No	Checklist item	Reported on page No
**Title and abstract**			
	1a	Identification as a pilot or feasibility randomised trial in the title	1
	1b	Structured summary of pilot trial design, methods, results, and conclusions (for specific guidance see CONSORT abstract extension for pilot trials)	2
**Introduction**			
Background and objectives	2a	Scientific background and explanation of rationale for future definitive trial, and reasons for randomised pilot trial	3
	2b	Specific objectives or research questions for pilot trial	3
**Methods**			
Trial design	3a	Description of pilot trial design (such as parallel, factorial) including allocation ratio	3
	3b	Important changes to methods after pilot trial commencement (such as eligibility criteria), with reasons	N/A
Participants	4a	Eligibility criteria for participants	4
	4b	Settings and locations where the data were collected	5
	4c	How participants were identified and consented	4
Interventions	5	The interventions for each group with sufficient details to allow replication, including how and when they were actually administered	2-5
Outcomes	6a	Completely defined prespecified assessments or measurements to address each pilot trial objective specified in 2b, including how and when they were assessed	4/5
	6b	Any changes to pilot trial assessments or measurements after the pilot trial commenced, with reasons	N/A
	6c	If applicable, prespecified criteria used to judge whether, or how, to proceed with future definitive trial	N/A

Sample size	7a	Rationale for numbers in the pilot trial	N/A
	7b	When applicable, explanation of any interim analyses and stopping guidelines	N/A
Randomisation:			
Sequence generation	8a	Method used to generate the random allocation sequence	4
	8b	Type of randomisation(s); details of any restriction (such as blocking and block size)	4/5
Allocation concealment mechanism	9	Mechanism used to implement the random allocation sequence (such as sequentially numbered containers), describing any steps taken to conceal the sequence until interventions were assigned	4
Implementation	10	Who generated the random allocation sequence, who enrolled participants, and who assigned participants to interventions	4/5
Blinding	11a	If done, who was blinded after assignment to interventions (for example, participants, care providers, those assessing outcomes) and how	4/5
	11b	If relevant, description of the similarity of interventions	4/5
Statistical methods	12	Methods used to address each pilot trial objective whether qualitative or quantitative	4/5
**Results**			
Participant flow (a diagram is strongly recommended)	13a	For each group, the numbers of participants who were approached and/or assessed for eligibility, randomly assigned, received intended treatment, and were assessed for each objective	6
	13b	For each group, losses and exclusions after randomisation, together with reasons	6
Recruitment	14a	Dates defining the periods of recruitment and follow-up	6
	14b	Why the pilot trial ended or was stopped	N/A
Baseline data	15	A table showing baseline demographic and clinical characteristics for each group	10
Numbers analysed	16	For each objective, number of participants (denominator) included in each analysis. If relevant, these numbers should be by randomised group	6

Outcomes and estimation	17	For each objective, results including expressions of uncertainty (such as 95% confidence interval) for any estimates. If relevant, these results should be by randomised group	6
Ancillary analyses	18	Results of any other analyses performed that could be used to inform the future definitive trial	6/7
Harms	19	All important harms or unintended effects in each group (for specific guidance see CONSORT for harms)	N/A
	19a	If relevant, other important unintended consequences	6/7
**Discussion**			
Limitations	20	Pilot trial limitations, addressing sources of potential bias and remaining uncertainty about feasibility	8
Generalisability	21	Generalisability (applicability) of pilot trial methods and findings to future definitive trial and other studies	8
Interpretation	22	Interpretation consistent with pilot trial objectives and findings, balancing potential benefits and harms, and considering other relevant evidence	8/9
	22a	Implications for progression from pilot to future definitive trial, including any proposed amendments	8/9
**Other information**			
Registration	23	Registration number for pilot trial and name of trial registry	4
Protocol	24	Where the pilot trial protocol can be accessed, if available	4
Funding	25	Sources of funding and other support (such as supply of drugs), role of funders	1
	26	Ethical approval or approval by research review committee, confirmed with reference number	4

Citation: Eldridge SM, Chan CL, Campbell MJ, Bond CM, Hopewell S, Thabane L, et al. CONSORT 2010 statement: extension to randomised pilot and feasibility trials. BMJ. 2016;355. This is an Open Access article distributed in accordance with the terms of the Creative Commons Attribution (CC BY 3.0) license (http://creativecommons.org/licenses/by/3.0/), which permits others to distribute, remix, adapt and build upon this work, for commercial use, provided the original work is properly cited.

The TIDieR (Template for Intervention Description and Replication) Checklist*:

Item number	Item	Where located**	
		**Primary paper (page or appendix number)**	**Other† (details)**
**1**	**BRIEF NAME**<br>Provide the name or a phrase that describes the intervention	**2**	
**2**	**WHY**<br>Describe any rationale, theory, or goal of the elements essential to the intervention.	**2,3**	
**3**	**WHAT**<br>Materials: Describe any physical or informational materials used in the intervention, including those provided to participants or used in intervention delivery or in training of intervention providers. Provide information on where the materials can be accessed (e.g. online appendix, URL).	**2-4**	10.3389/fgstr.2024.1359002
**4**	Procedures: Describe each of the procedures, activities, and/or processes used in the intervention, including any enabling or support activities	**3,4**	
**5**	**WHO PROVIDED**<br>For each category of intervention provider (e.g. psychologist, nursing assistant), describe their expertise, background and any specific training given	**2,3**	
**6**	**HOW**<br>Describe the modes of delivery (e.g. face-to-face or by some other mechanism, such as internet or telephone) of the intervention and whether it was provided individually or in a group	**2-4**	
**7**	**WHERE**<br>Describe the type(s) of location(s) where the intervention occurred, including any necessary infrastructure or relevant features.	**2-5**	
**8**	**WHEN and HOW MUCH**<br>Describe the number of times the intervention was delivered and over what period of time including the number of sessions, their schedule, and their duration, intensity or dose.	**2-5**	
**9**	**TAILORING**<br>If the intervention was planned to be personalised, titrated or adapted, then describe what, why, when, and how	**N/A**	
**10**	**MODIFICATIONS**<br>If the intervention was modified during the course of the study, describe the changes (what, why, when, and how)	**N/A**	
**11**	**HOW WELL**<br>Planned: If intervention adherence or fidelity was assessed, describe how and by whom, and if any strategies were used to maintain or improve fidelity, describe them	**N/A**	
**12‡**	Actual: If intervention adherence or fidelity was assessed, describe the extent to which the intervention was delivered as planned.	**N/A**	

** **Authors** - use N/A if an item is not applicable for the intervention being described. **Reviewers** – use '?' if information about the element is not reported/not sufficiently reported.

† If the information is not provided in the primary paper, give details of where this information is available. This may include locations such as a published protocol or other published papers (provide citation details) or a website (provide the URL).

‡ If completing the TIDieR checklist for a protocol, these items are not relevant to the protocol and cannot be described until the study is complete.

* We strongly recommend using this checklist in conjunction with the TIDieR guide (see *BMJ* 2014;348:g1687) which contains an explanation and elaboration for each item.

* The focus of TIDieR is on reporting details of the intervention elements (and where relevant, comparison elements) of a study. Other elements and methodological features of studies are covered by other reporting statements and checklists and have not been duplicated as part of the TIDieR checklist. When a **randomised trial** is being reported, the TIDieR checklist should be used in conjunction with the CONSORT statement (see www.consort-statement.org) as an extension of **Item 5 of the CONSORT 2010 Statement.** When a **clinical trial protocol** is being reported, the TIDieR checklist should be used in conjunction with the SPIRIT statement as an extension of **Item 11 of the SPIRIT 2013 Statement** (see www.spirit-statement.org). For alternate study designs, TIDieR can be used in conjunction with the appropriate checklist for that study design (see www.equator-network.org).
